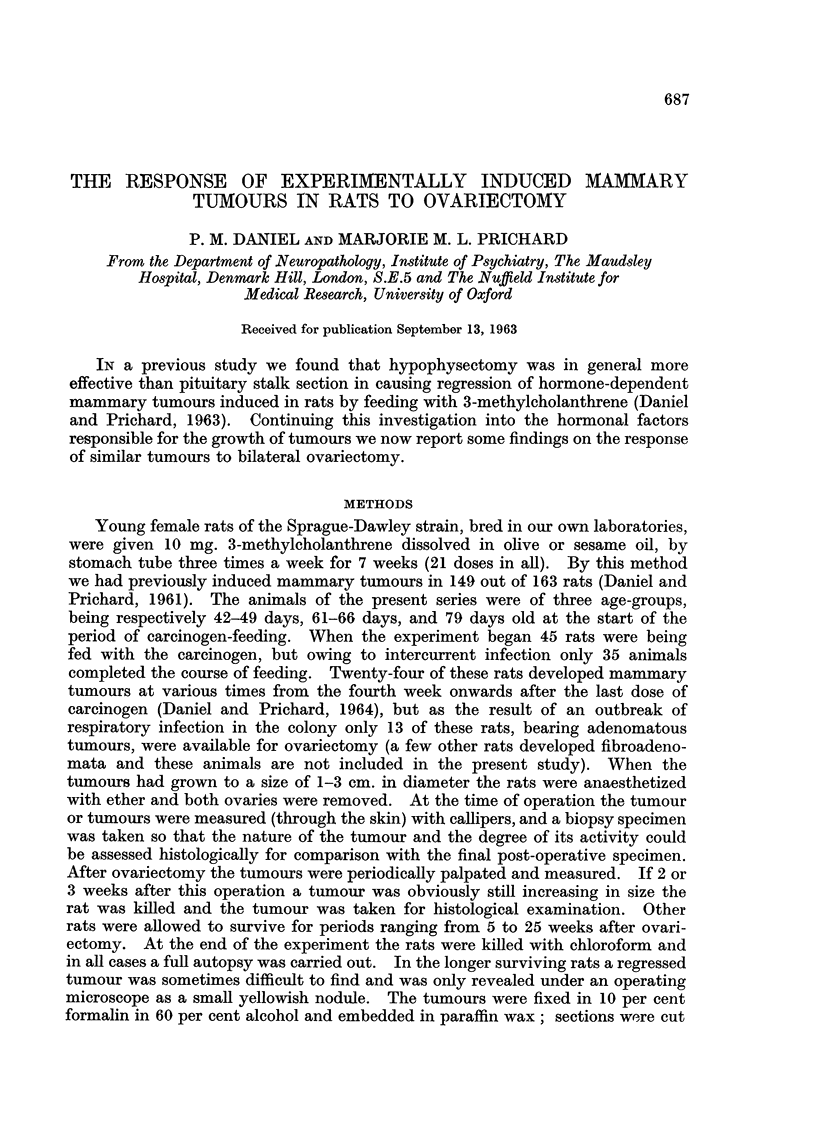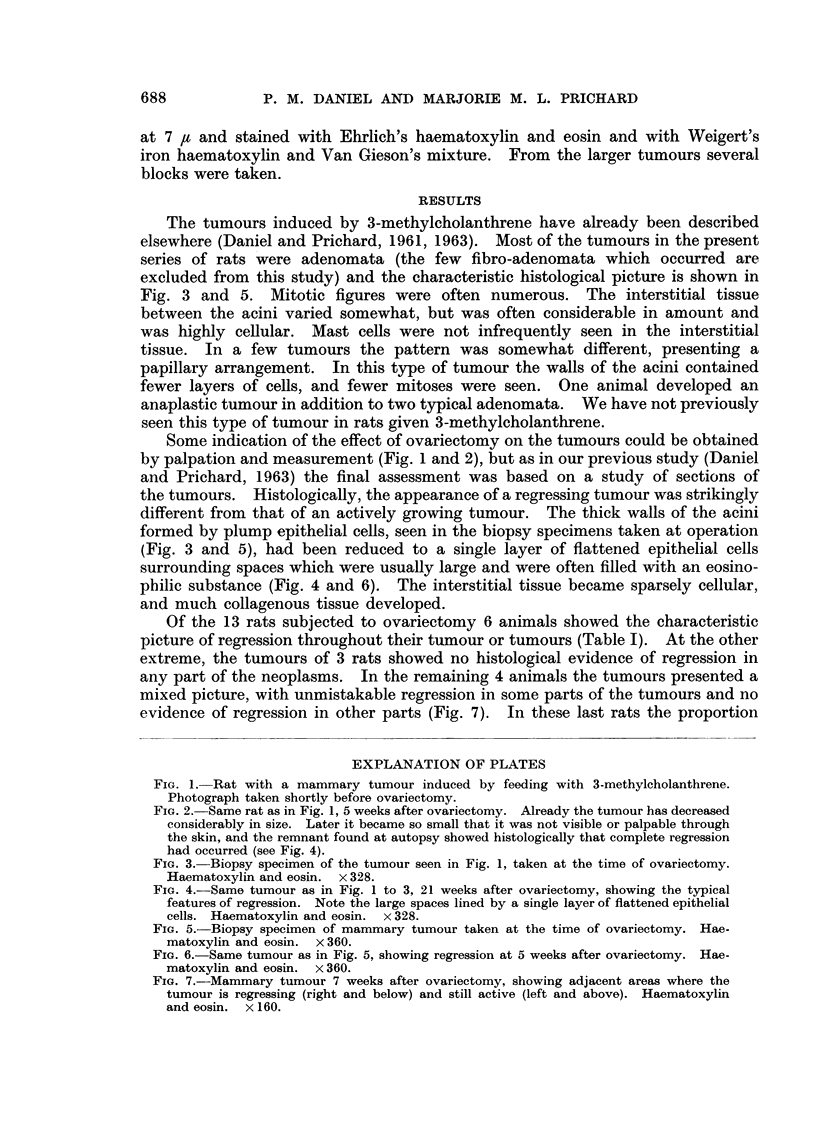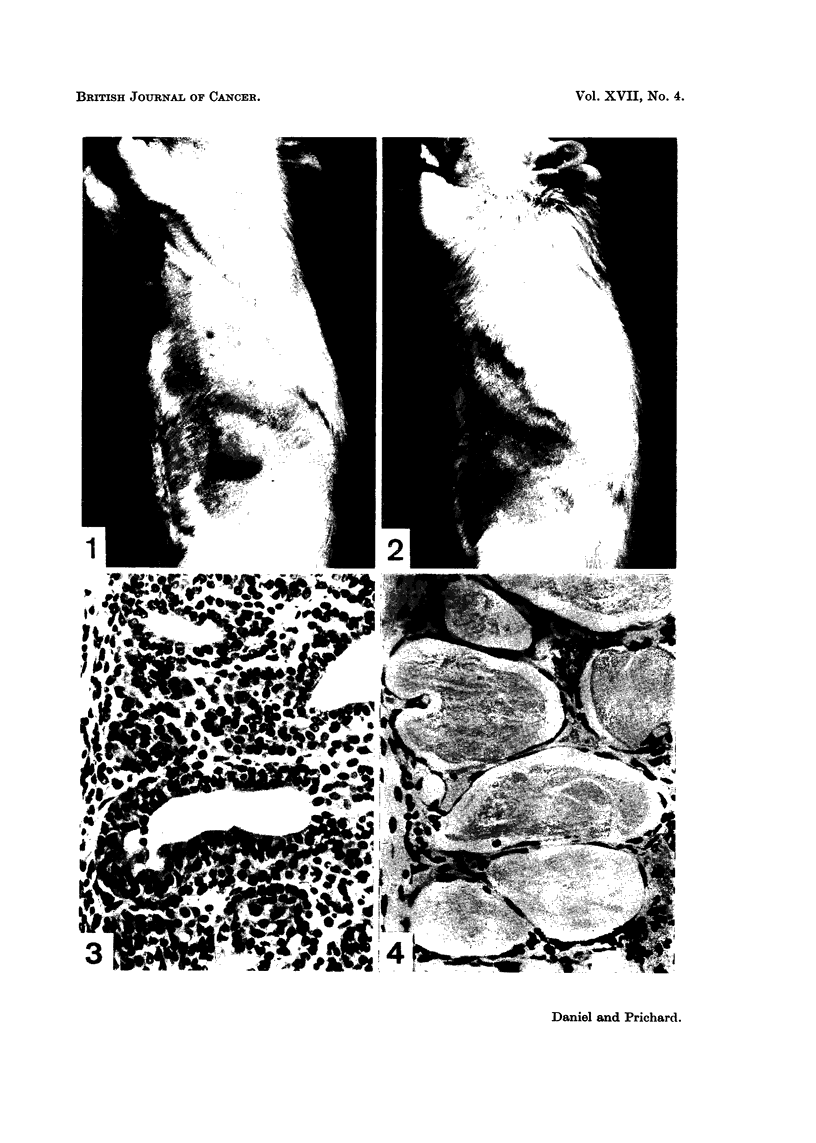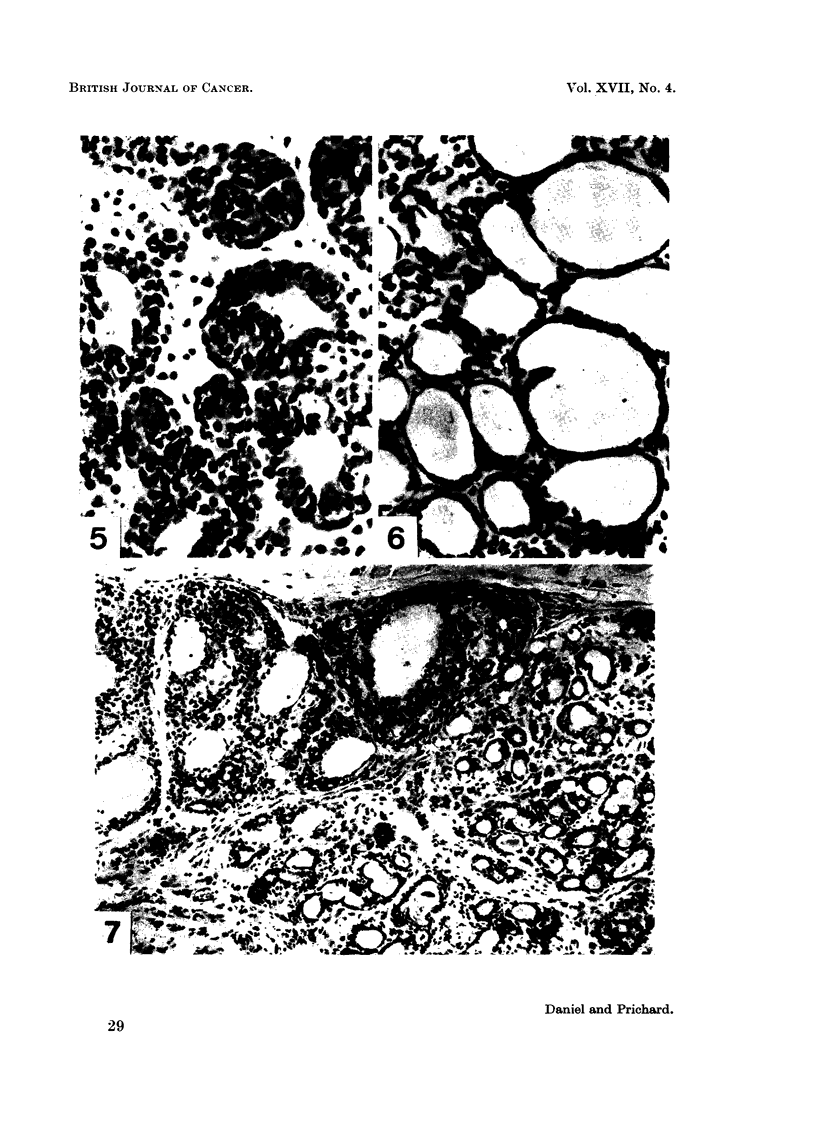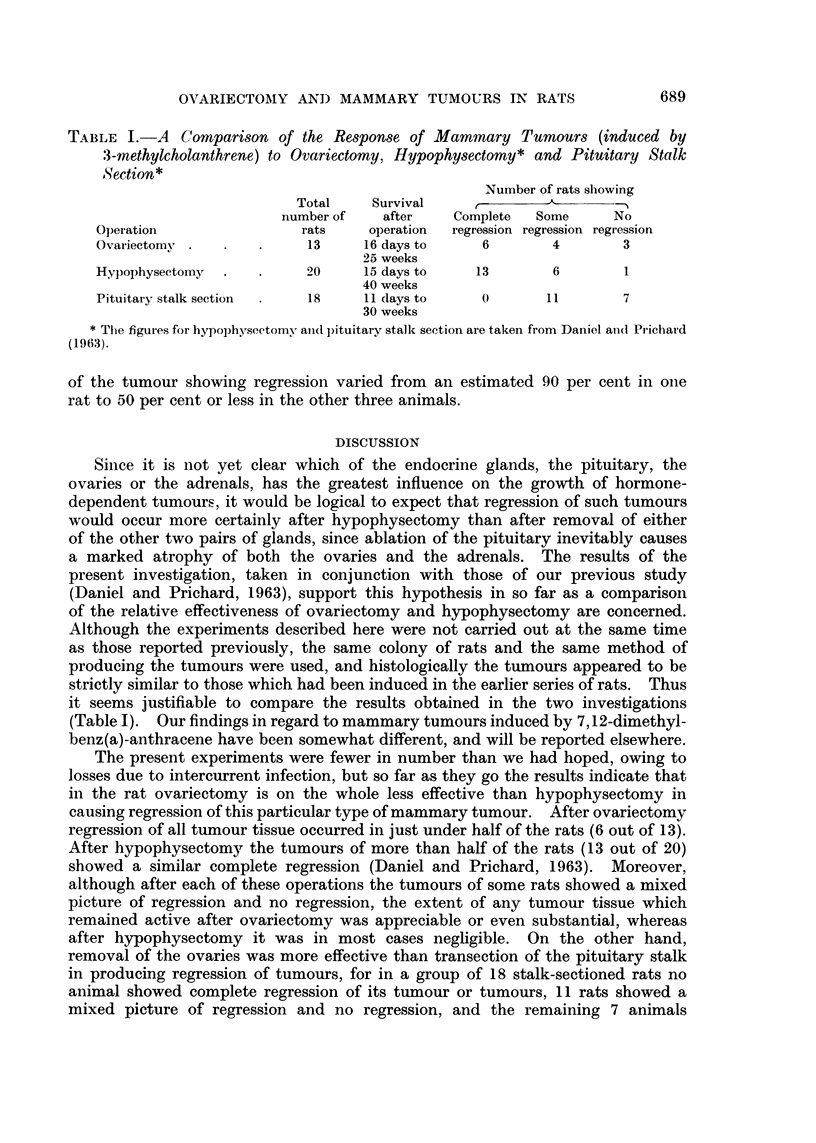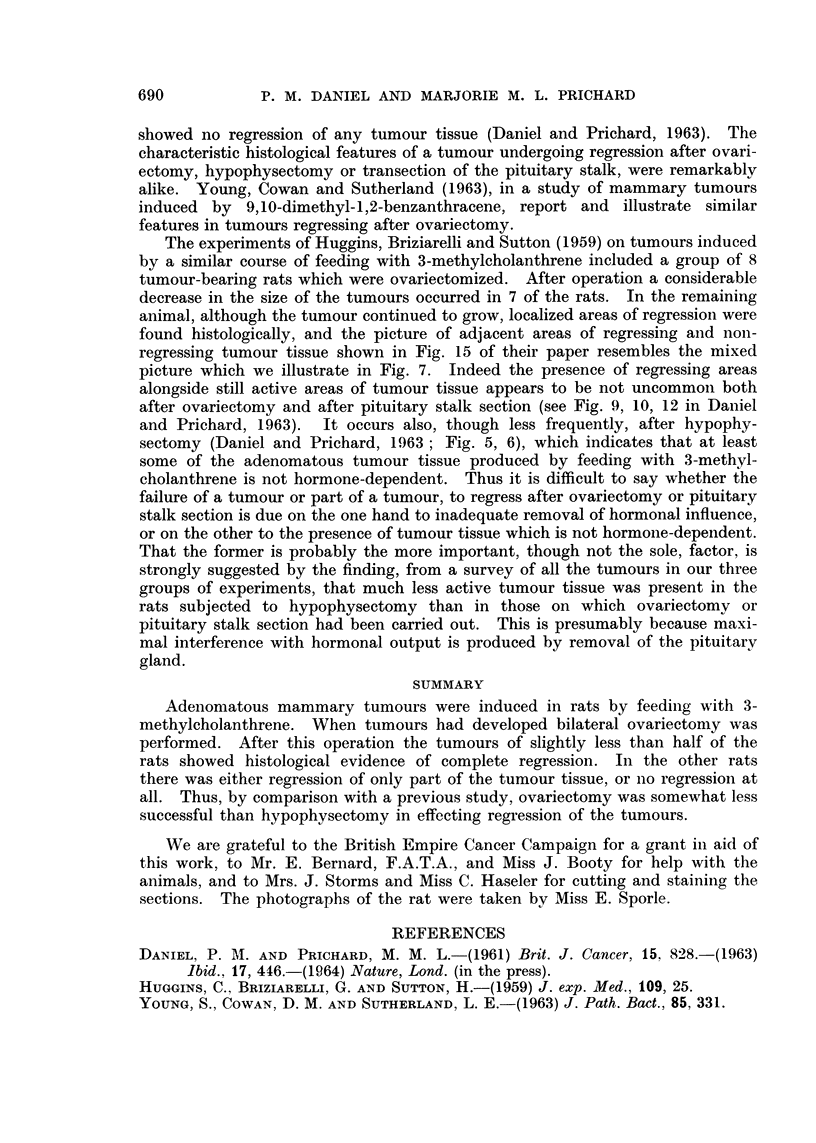# The Response of Experimentally Induced Mammary Tumours in Rats to Ovariectomy

**DOI:** 10.1038/bjc.1963.87

**Published:** 1963-12

**Authors:** P. M. Daniel, Marjorie M. L. Prichard

## Abstract

**Images:**


### THE RESPONSE OF EXPERIMENTALLY INDUCED MAMMARY TUMOURS IN RATS TO OVARIECTOMY

P. M. DANIEL AND MARJORIE M. L. PRICHARD

*From the Department of Neuropathology, Institute of Psychiatry, The Maudsley Hospital, Denmark Hill, London, S.E.5 and The Nuffield Institute for Medical Research, University of Oxford*

Received for publication September 13, 1963

IN a previous study we found that hypophysectomy was in general more effective than pituitary stalk section in causing regression of hormone-dependent mammary tumours induced in rats by feeding with 3-methylcholanthrene (Daniel and Prichard, 1963). Continuing this investigation into the hormonal factors responsible for the growth of tumours we now report some findings on the response of similar tumours to bilateral ovariectomy.

### METHODS

Young female rats of the Sprague-Dawley strain, bred in our own laboratories, were given 10 mg. 3-methylcholanthrene dissolved in olive or sesame oil, by stomach tube three times a week for 7 weeks (21 doses in all). By this method we had previously induced mammary tumours in 149 out of 163 rats (Daniel and Prichard, 1961). The animals of the present series were of three age-groups, being respectively 42–49 days, 61–66 days, and 79 days old at the start of the period of carcinogen-feeding. When the experiment began 45 rats were being fed with the carcinogen, but owing to intercurrent infection only 35 animals completed the course of feeding. Twenty-four of these rats developed mammary tumours at various times from the fourth week onwards after the last dose of carcinogen (Daniel and Prichard, 1964), but as the result of an outbreak of respiratory infection in the colony only 13 of these rats, bearing adenomatous tumours, were available for ovariectomy (a few other rats developed fibroadenomata and these animals are not included in the present study). When the tumours had grown to a size of 1–3 cm. in diameter the rats were anaesthetized with ether and both ovaries were removed. At the time of operation the tumour or tumours were measured (through the skin) with callipers, and a biopsy specimen was taken so that the nature of the tumour and the degree of its activity could be assessed histologically for comparison with the final post-operative specimen. After ovariectomy the tumours were periodically palpated and measured. If 2 or 3 weeks after this operation a tumour was obviously still increasing in size the rat was killed and the tumour was taken for histological examination. Other rats were allowed to survive for periods ranging from 5 to 25 weeks after ovariectomy. At the end of the experiment the rats were killed with chloroform and in all cases a full autopsy was carried out. In the longer surviving rats a regressed tumour was sometimes difficult to find and was only revealed under an operating microscope as a small yellowish nodule. The tumours were fixed in 10 per cent formalin in 60 per cent alcohol and embedded in paraffin wax ; sections were cut at 7 $\mu$ and stained with Ehrlich's haematoxylin and eosin and with Weigert's iron haematoxylin and Van Gieson's mixture. From the larger tumours several blocks were taken.

### RESULTS

The tumours induced by 3-methylcholanthrene have already been described elsewhere (Daniel and Prichard, 1961, 1963). Most of the tumours in the present series of rats were adenomata (the few fibro-adenomata which occurred are excluded from this study) and the characteristic histological picture is shown in Fig. 3 and 5. Mitotic figures were often numerous. The interstitial tissue between the acini varied somewhat, but was often considerable in amount and was highly cellular. Mast cells were not infrequently seen in the interstitial tissue. In a few tumours the pattern was somewhat different, presenting a papillary arrangement. In this type of tumour the walls of the acini contained fewer layers of cells, and fewer mitoses were seen. One animal developed an anaplastic tumour in addition to two typical adenomata. We have not previously seen this type of tumour in rats given 3-methylcholanthrene.

Some indication of the effect of ovariectomy on the tumours could be obtained by palpation and measurement (Fig. 1 and 2), but as in our previous study (Daniel and Prichard, 1963) the final assessment was based on a study of sections of the tumours. Histologically, the appearance of a regressing tumour was strikingly different from that of an actively growing tumour. The thick walls of the acini formed by plump epithelial cells, seen in the biopsy specimens taken at operation (Fig. 3 and 5), had been reduced to a single layer of flattened epithelial cells surrounding spaces which were usually large and were often filled with an eosinophilic substance (Fig. 4 and 6). The interstitial tissue became sparsely cellular, and much collagenous tissue developed.

Of the 13 rats subjected to ovariectomy 6 animals showed the characteristic picture of regression throughout their tumour or tumours (Table I). At the other extreme, the tumours of 3 rats showed no histological evidence of regression in any part of the neoplasms. In the remaining 4 animals the tumours presented a mixed picture, with unmistakable regression in some parts of the tumours and no evidence of regression in other parts (Fig. 7). In these last rats the proportion

---

### EXPLANATION OF PLATES

FIG. 1.—Rat with a mammary tumour induced by feeding with 3-methylcholanthrene. Photograph taken shortly before ovariectomy.

FIG. 2.—Same rat as in Fig. 1, 5 weeks after ovariectomy. Already the tumour has decreased considerably in size. Later it became so small that it was not visible or palpable through the skin, and the remnant found at autopsy showed histologically that complete regression had occurred (see Fig. 4).

FIG. 3.—Biopsy specimen of the tumour seen in Fig. 1, taken at the time of ovariectomy. Haematoxylin and eosin. ×328.

FIG. 4.—Same tumour as in Fig. 1 to 3, 21 weeks after ovariectomy, showing the typical features of regression. Note the large spaces lined by a single layer of flattened epithelial cells. Haematoxylin and eosin. ×328.

FIG. 5.—Biopsy specimen of mammary tumour taken at the time of ovariectomy. Haematoxylin and eosin. ×360.

FIG. 6.—Same tumour as in Fig. 5, showing regression at 5 weeks after ovariectomy. Haematoxylin and eosin. ×360.

FIG. 7.—Mammary tumour 7 weeks after ovariectomy, showing adjacent areas where the tumour is regressing (right and below) and still active (left and above). Haematoxylin and eosin. ×160.

1

2

3

4

5

6

7

TABLE I.—*A Comparison of the Response of Mammary Tumours (induced by 3-methylcholanthrene) to Ovariectomy, Hypophysectomy* and Pituitary Stalk Section**

| | | | Number of rats showing | | |
|---|---|---|---|---|---|
| Operation | Total number of rats | Survival after operation | Complete regression | Some regression | No regression |
| Ovariectomy . . . | 13 | 16 days to 25 weeks | 6 | 4 | 3 |
| Hypophysectomy . . | 20 | 15 days to 40 weeks | 13 | 6 | 1 |
| Pituitary stalk section . | 18 | 11 days to 30 weeks | 0 | 11 | 7 |

* The figures for hypophysectomy and pituitary stalk section are taken from Daniel and Prichard (1963).

of the tumour showing regression varied from an estimated 90 per cent in one rat to 50 per cent or less in the other three animals.

### DISCUSSION

Since it is not yet clear which of the endocrine glands, the pituitary, the ovaries or the adrenals, has the greatest influence on the growth of hormone-dependent tumours, it would be logical to expect that regression of such tumours would occur more certainly after hypophysectomy than after removal of either of the other two pairs of glands, since ablation of the pituitary inevitably causes a marked atrophy of both the ovaries and the adrenals. The results of the present investigation, taken in conjunction with those of our previous study (Daniel and Prichard, 1963), support this hypothesis in so far as a comparison of the relative effectiveness of ovariectomy and hypophysectomy are concerned. Although the experiments described here were not carried out at the same time as those reported previously, the same colony of rats and the same method of producing the tumours were used, and histologically the tumours appeared to be strictly similar to those which had been induced in the earlier series of rats. Thus it seems justifiable to compare the results obtained in the two investigations (Table I). Our findings in regard to mammary tumours induced by 7,12-dimethylbenz(a)-anthracene have been somewhat different, and will be reported elsewhere.

The present experiments were fewer in number than we had hoped, owing to losses due to intercurrent infection, but so far as they go the results indicate that in the rat ovariectomy is on the whole less effective than hypophysectomy in causing regression of this particular type of mammary tumour. After ovariectomy regression of all tumour tissue occurred in just under half of the rats (6 out of 13). After hypophysectomy the tumours of more than half of the rats (13 out of 20) showed a similar complete regression (Daniel and Prichard, 1963). Moreover, although after each of these operations the tumours of some rats showed a mixed picture of regression and no regression, the extent of any tumour tissue which remained active after ovariectomy was appreciable or even substantial, whereas after hypophysectomy it was in most cases negligible. On the other hand, removal of the ovaries was more effective than transection of the pituitary stalk in producing regression of tumours, for in a group of 18 stalk-sectioned rats no animal showed complete regression of its tumour or tumours, 11 rats showed a mixed picture of regression and no regression, and the remaining 7 animals showed no regression of any tumour tissue (Daniel and Prichard, 1963). The characteristic histological features of a tumour undergoing regression after ovariectomy, hypophysectomy or transection of the pituitary stalk, were remarkably alike. Young, Cowan and Sutherland (1963), in a study of mammary tumours induced by 9,10-dimethyl-1,2-benzanthracene, report and illustrate similar features in tumours regressing after ovariectomy.

The experiments of Huggins, Briziarelli and Sutton (1959) on tumours induced by a similar course of feeding with 3-methylcholanthrene included a group of 8 tumour-bearing rats which were ovariectomized. After operation a considerable decrease in the size of the tumours occurred in 7 of the rats. In the remaining animal, although the tumour continued to grow, localized areas of regression were found histologically, and the picture of adjacent areas of regressing and non-regressing tumour tissue shown in Fig. 15 of their paper resembles the mixed picture which we illustrate in Fig. 7. Indeed the presence of regressing areas alongside still active areas of tumour tissue appears to be not uncommon both after ovariectomy and after pituitary stalk section (see Fig. 9, 10, 12 in Daniel and Prichard, 1963). It occurs also, though less frequently, after hypophysectomy (Daniel and Prichard, 1963 ; Fig. 5, 6), which indicates that at least some of the adenomatous tumour tissue produced by feeding with 3-methylcholanthrene is not hormone-dependent. Thus it is difficult to say whether the failure of a tumour or part of a tumour, to regress after ovariectomy or pituitary stalk section is due on the one hand to inadequate removal of hormonal influence, or on the other to the presence of tumour tissue which is not hormone-dependent. That the former is probably the more important, though not the sole, factor, is strongly suggested by the finding, from a survey of all the tumours in our three groups of experiments, that much less active tumour tissue was present in the rats subjected to hypophysectomy than in those on which ovariectomy or pituitary stalk section had been carried out. This is presumably because maximal interference with hormonal output is produced by removal of the pituitary gland.

### SUMMARY

Adenomatous mammary tumours were induced in rats by feeding with 3-methylcholanthrene. When tumours had developed bilateral ovariectomy was performed. After this operation the tumours of slightly less than half of the rats showed histological evidence of complete regression. In the other rats there was either regression of only part of the tumour tissue, or no regression at all. Thus, by comparison with a previous study, ovariectomy was somewhat less successful than hypophysectomy in effecting regression of the tumours.

We are grateful to the British Empire Cancer Campaign for a grant in aid of this work, to Mr. E. Bernard, F.A.T.A., and Miss J. Booty for help with the animals, and to Mrs. J. Storms and Miss C. Haseler for cutting and staining the sections. The photographs of the rat were taken by Miss E. Sporle.

### REFERENCES

Daniel, P. M. and Prichard, M. M. L.—(1961) *Brit. J. Cancer*, **15**, 828.—(1963) *Ibid.*, **17**, 446.—(1964) *Nature, Lond.* (in the press).

Huggins, C., Briziarelli, G. and Sutton, H.—(1959) *J. exp. Med.*, **109**, 25.

Young, S., Cowan, D. M. and Sutherland, L. E.—(1963) *J. Path. Bact.*, **85**, 331.